# Modelling the high-voltage grid using open data for Europe and beyond

**DOI:** 10.1038/s41597-025-04550-7

**Published:** 2025-02-16

**Authors:** Bobby Xiong, Davide Fioriti, Fabian Neumann, Iegor Riepin, Tom Brown

**Affiliations:** 1 Department of Digital Transformation in Energy Systems, Institute of Energy Engineering, Technische Universität Berlin, Berlin, Germany; 2 Department of Energy Systems, Territory and Construction Engineering, Università di Pisa, Pisa, Italy

**Keywords:** Energy modelling, Energy grids and networks

## Abstract

This paper provides the background, methodology and validation for constructing a representation of the European high-voltage grid (AC lines from 220 to 750 kV and all DC lines) based on OpenStreetMap data. Grid components include commissioned substations, transmission lines and cables, transformers, and converters as well as technical parameters based on standard types. The data is provided as easy-to-access comma-separated values files which makes it suitable for model-independent, large-scale electricity and energy system modelling. For further ease-of-use, an interactive map is included to enable visual inspection. To assess the data quality, this paper compares the dataset with official statistics and representative model runs using PyPSA-Eur based on different electricity grid representations. The dataset and workflow are provided as part of PyPSA-Eur, an open-source, sector-coupled optimisation model of the European energy system. By integrating with the codebase for initiatives such as PyPSA-Earth, the benefits of this work of this work extend to the global context. The dataset is published under the Open Data Commons Open Database (ODbL 1.0) licence.

## Background & Summary

Energy system models are indispensable tools in today’s world in order to understand the complex interactions between energy sources, technologies, policies, and markets. They are used by researchers, industry and policy makers to enable informed decision-making in the transition to a net-zero energy system. However, conclusions drawn from such models are only as good as the underlying data and assumptions. Especially the representation of existing energy infrastructure, such as the electricity grid, can have a deciding impact on future investments derived from such models^1^. While Transmission System Operators (TSOs) have their own information on the high-voltage grid, this data is often not publicly available to the level of detail needed for academic research purposes. Official institutions like the European Network of Transmission System Operators for Electricity (ENTSO-E) provide an online map^2^ of the European high-voltage grid. There are, however, several limitations typical for these sources: (i) there is no underlying, topologically connected dataset, (ii) they are not released under an open licence, (iii) nor are they updated frequently, and (iv) their geographic detail is limited or highly stylised.

There are previous projects that have modelled the European high-voltage grid or its parts based on OSM data. Some institutions provide data for particular regions, however, all of them come with their own drawbacks: While the most trustworthy data comes from TSOs themselves, they are — with few regional exceptions^3^ — not georeferenced^4^ or do not cover the entirety of Europe. Datasets from previous academic projects^5–7^ are either very complex to reproduce or have not been updated for close to a decade. An overview of notable projects and datasets is listed in Table 6. Proven to be a reliable public data source, we make use of OSM to introduce a transparent workflow in order to create a representation of the European high-voltage grid. On the lack of updates of existing datasets, there are two main advantages of our work compared to previous initiatives. First, our approach uses the OSM Overpass turbo API^8^ that always allows to retrieve the latest OSM data. Second, the active OSM community, along with the large user base of PyPSA-Eur and its integration into automated workflows ensure frequent updates and validation. Debugging can be easily done with the help of the open source project OpenInfraMap^9^ and the interactive map included in our dataset. Both tools render the electricity infrastructure in a JavaScript-based map which can be accessed with any modern browser. Finally, the entire workflow is developed in Python and may hence be more accessible than other implementations which require external dependencies (e.g. SQL databases, commercial software, Java)^10^.

**Table 1:** Key tags and parameters for AC power lines and cables.

Tag	Data type	Example
cables	numeric	9
circuits	numeric	3
frequency	numeric	50 Hz
power	string	line
voltage	numeric	380000 V

**Table 2:** Illustrative example of AC lines and cables input data.

line id	cables	circuits	frequency (Hz)	type	voltage (V)
way/1		2	50	cable	380000
relation/1	3		50	cable	380000
way/3	9	1;2	50	line	380000; 220000
way/4	9	3	50; 50	line	380000; 220000
way/5	8		50	line	110000; 220000
way/6			50	cable	300000

**Table 3:** Illustrative example of AC lines and cables after cleaning. Changes highlighted in bold.

line id	circuits	frequency (Hz)	type	voltage (V)
way/1	2	50	cable	380000
relation/1	**1**	50	cable	380000
way/3-1	**1**	50	line	**380000**
way/3-2	**2**	50	line	**220000**
way/4-1	**1**	**50**	line	**380000**
way/4-2	**1**	**50**	line	**220000**
way/5-1	**1**	50	line	**110000 (removed)**
way/5-2	**1**	50	line	**220000**
way/6	**1**	50	cable	300000

**Table 4:** Comparison of key result metrics between ENTSO-E map and OSM-based transmission grid. CO_2_ price: 100 €/t.

	System costs (bn. €)	CAPEX (bn. €)	OPEX (bn. €)	Curtailment (TWh)	Generation (TWh)
GridKit	319.28	283.47	35.81	2749.41	3119.82
OSM	318.19	283.09	35.1	2742.26	3118.5
Delta (Percent)	−0.34	−0.13	−1.99	−0.26	−0.04

Compared to previous implementations in the global modal PyPSA-Earth^11^, we significantly improve the work in speed and data quality by taking advantage of the topological, electrical and geographical information available for Europe in OSM. Given the generic structure of the developed workflow, it can be easily applied to other regions and fed back to the global PyPSA-Earth project. However, the output will directly depend on the OSM data quality for a particular region (e.g. whether data on the substation’s geometric footprint is available). To fill in missing data, we introduce cleaning process that yields a representation of the European high-voltage grid. We benchmark the processed data against country-level statistics provided by ENTSO-E, concluding that OSM data coverage of the European high-voltage grid is high or even close to complete. These improvements will also contribute to the quality of transmission grids modelled on a global scale in PyPSA-Earth.

## Methods

PyPSA-Eur is a spatially and temporally highly resolved, open-source, sector-coupled linear optimisation model that covers the European continent^12^. The model is built on top of the open-source toolbox PyPSA^13^ and is suited for operational as well as expansion studies (transmission, generation, and storages). The model includes a stock of existing power plants (processed with the tool powerplantmatching^14^) as well as renewable potentials and availability time series (processed with atlite^15^). Throughout the last decade, PyPSA-Eur has gained a large user base from academia, industry, and policy makers alike and has been used in a variety of studies^16–23^. Other open-source models exist — notable and widely-used ones include the *oemof*^24^ and *OSeMOSYS Global*^25^. However, both models lack the detailed geographical as well as electrical representation of the transmission grid that PyPSA-Eur provides. While the open European model *Euro-Calliope* does provide a high spatial resolution, it models the grid with net transfer capacities^26^.

With the integration into PyPSA-Eur, we also enable compatibility with additional functions already implemented into the model, such as, but not limited to, the option to enable dynamic line rating^27^ and the inclusion of projects under planning (e.g. European Ten-Year Network Development Plan^28^ and the German Network Development Plan^29^).

**Fig. 1:**
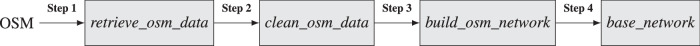
Process diagram depicting the creation of the European high-voltage grid from OSM data, implemented through individual Snakemake rules.

**Fig. 2:**
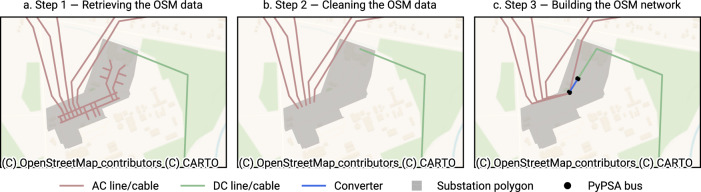
Illustration of the steps to create a PyPSA-ready network from OSM data. Note that Step 4 does not make changes to the topology and is hence omitted from this illustration.

PyPSA-Eur is managed by a workflow management system called Snakemake^30^. Its modular structure enables the addition of new model functionalities and data sources, these can then be toggled using a configuration file. We split the construction of the high-voltage grid into four steps and add them into the existing workflow (see Fig. 1). We also use the model for validation purposes (see section Technical Validation). While the dataset and its reconstruction is built into PyPSA-Eur, it has the potential to be used in other energy system models, too. For this purpose, we release the entire dataset, including the original and mapped technical parameters. To obtain a functioning, topologically connected representation of the European high-voltage grid based on OSM data, we take the following steps (see Fig. 2 for an application to an example region).

### Step 1 — Retrieving the OSM data

In OSM, geographical data is stored in ‘nodes’, ‘ways’, and ‘relations’. Being the simplest data type, nodes are defined by coordinates and associated parameters. Ways are geometric line strings that connect a set of nodes. Relations can contain nodes, ways, a combination of either or other relations^7^. For the purpose of this work, we extract ways for obtaining the outline of substations. For AC lines we use both ways and relations data to build the dataset. To harness the highest data quality, we use relational data whenever available. To avoid duplicates in the representation of an AC line, all members (ways) of a relation are removed from the dataset. This hybrid approach allows us to make use of relations whenever they are complete and exist, and fall back to ways if relations are incomplete or missing.

To obtain DC projects (usually cables, hereafter referred to as DC links) we use relations. There are two main reasons for this differentiation: (i) relations are the more complex data type, coverage for AC lines and cables is still scarce for Europe. For DC links, given that there are fewer of them in Europe, we have checked each project individually using the latest ENTSO-E map^2^ and compared their technical parameters (e.g. nominal capacity) with previous datasets^10,13^. Based on this, we consider the coverage of commissioned DC links complete (see Table 7); (ii) Some DC links contain multiple components, accessing relations allows us to efficiently aggregate and simplify the links for the purpose of static energy system modelling.

First, we retrieve the raw data using the Overpass turbo API^8^ (Fig. 2a). Specifically, we query the OSM database for electricity grid related features (see Table 5). Note that Overpass turbo has a limit on the size and number of requests for each query and provides the API under a fair use policy. To minimise unnecessary load and enable data reusability, we provide a prepared transmission grid for download via Zenodo (0.6 is the latest version at the time of publication)^31^.

### Step 2 — Cleaning the OSM data

While OSM provides a rich dataset, it is not directly usable for energy system modelling. Next to geospatial coordinates, OSM includes tags that provide feature-specific additional information. Depending on the individual feature, data may however contain noise, be incomplete, or inconsistent. After importing the retrieved raw data into pandas dataframes^32^, we apply a series of steps including heuristics to clean and fill in the missing information (see example in Table 2). We then use the power of geopandas^33^ to perform geospatial operations (including but not limited to spatial joins, intersections, buffering, etc.).

#### Substations and transformers

To obtain the set of substations, we filter for substations with a voltage level within the scope of interest, i.e., between AC 220 and 750 kV. Where available, we extract the polygon shape of the substations, stored in the element’s geometry. This allows us to differentiate between internal and external grid components. Note that information on transformers is not extracted from OSM, as (i) we cannot adequately evaluate their coverage and (ii) this data is not sufficient to create a topologically connected network. Instead, we use a needs-based approach, i.e., adding a single transformer between buses of different voltages within the perimeter of the same substation. The nominal capacity of each transformer is determined by the maximum aggregated capacity of connected AC lines and cables on either side. This is in line with previous approaches to obtain the ENTSO-E map based transmission grid^12^.

#### AC lines and cables

In a first step, we clean the tag columns to only contain the correct data type and unit, as shown in Table 1 for AC power lines and cables. The minimum parameters that need to be given for a particular line or cable are ‘voltage’ (in) and ‘power’ (string: ‘line’ or ‘cable’).

We filter for the entries with a voltage level including and above AC 200 kV. While data for the mid- to low-voltage grid is also partially available in OSM, public statistics are scarce, making validation of such data difficult. As not all entries contain clean or complete data, we make heuristic assumptions to fill in the gaps, as illustrated in Tables 2 and 3.

For each line or cable we use the most specific information available that the data provides. In a three-phase AC high-voltage system, we assume three cables to form an AC circuit (e.g. way/2)^34^. If a way contains multiple data points split by semicolons (i.e., transmission lines sharing overhead line routes), we split the entries into individual lines, accordingly. In this process, we preserve the original OSM identifier and its associated geometries. We add a numbered suffix after the split to maintain unique line ids (e.g. way/3 becomes way/3-1 and way/3-2). The given electric parameters are mapped according to the semicolon splits. In some cases however, where the number of data points across columns is not equal (e.g. way/4 and way/5), we make the following assumption: we take the floor of the number of circuits divided by the number of entries in the voltage column. In the absence of better information, this may lead to an underestimation of the real number of circuits.

If no information on cables or circuits is available, we assume a single circuit, provided that a voltage level is given (e.g. way/6). Finally, we remove all ways representing bus bars and lines fully contained within substation outline (Fig. 2b), as they are considered internal elements and do not provide additional information for static analyses.

### DC links and converters

Due to their distinct electrical properties, we treat DC links differently from AC lines and cables. To avoid double counting, we remove all DC links from the original way queries. Instead, we query the OSM database for relations that contain DC links. As data on DC projects is widely available and because there are fewer of them^2,35^, we contribute to the OSM database by adding missing parameters such as the nominal rating and voltage level. This signifies the ease of data improvements with OSM for the benefit of all. To trace future DC projects through our workflow, the following tags are required: ‘route’ = ‘power’, ‘frequency’ = 0, and ‘rating’ in ‘X MW’ format. DC components need to be correctly linked in the OSM database as member (either ‘cable’ or ‘line’) of the parent relation.

**Fig. 3:**
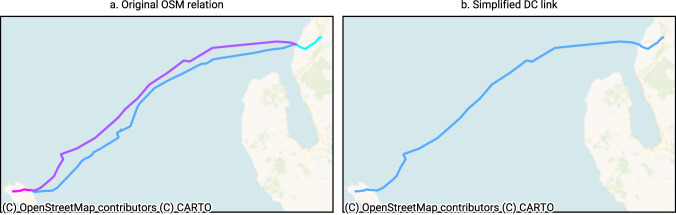
Example — Simplification of a DC link relation: Moyle interconnector (Scotland - Northern Ireland), OSM relation ID 6914309. Different colors show the four segments, i.e., ways that compose the OSM relation.

Note that some relations contain multiple DC link segments or components (e.g. converter stations or grounding), these are simplified into a single line with the sum of their nominal ratings. Figure 3 shows an example of how the Moyle interconnector from Northern Ireland to Scotland (Fig. 3a) is simplified (Fig. 3b). In this simplification, we preserve original end points of the DC link and the longest connected path. In PyPSA, converters are modelled as links connecting two buses. By analogy with how transformers are added to the network ex post, we connect the terminals of the DC link to their associated or nearest AC bus in the transmission grid. As such, we guarantee that DC links are always topologically connected.

### Step 3 — Building the OSM network

**Fig. 4:**
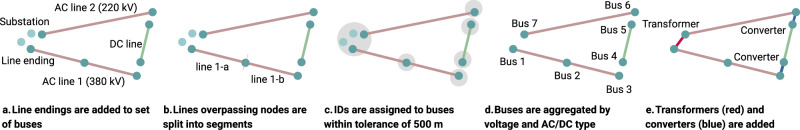
Illustration of building a topologically connected grid.

After having obtained a cleaned dataset, we need to ensure that the grid components are topologically and electrically connected. For this purpose, we propose a complete graph-based structure, i.e., buses/substations are connected by AC and DC lines, as well as converters and transformers. We introduce the following procedure, illustrated in Fig. 4.

**Fig. 5:**
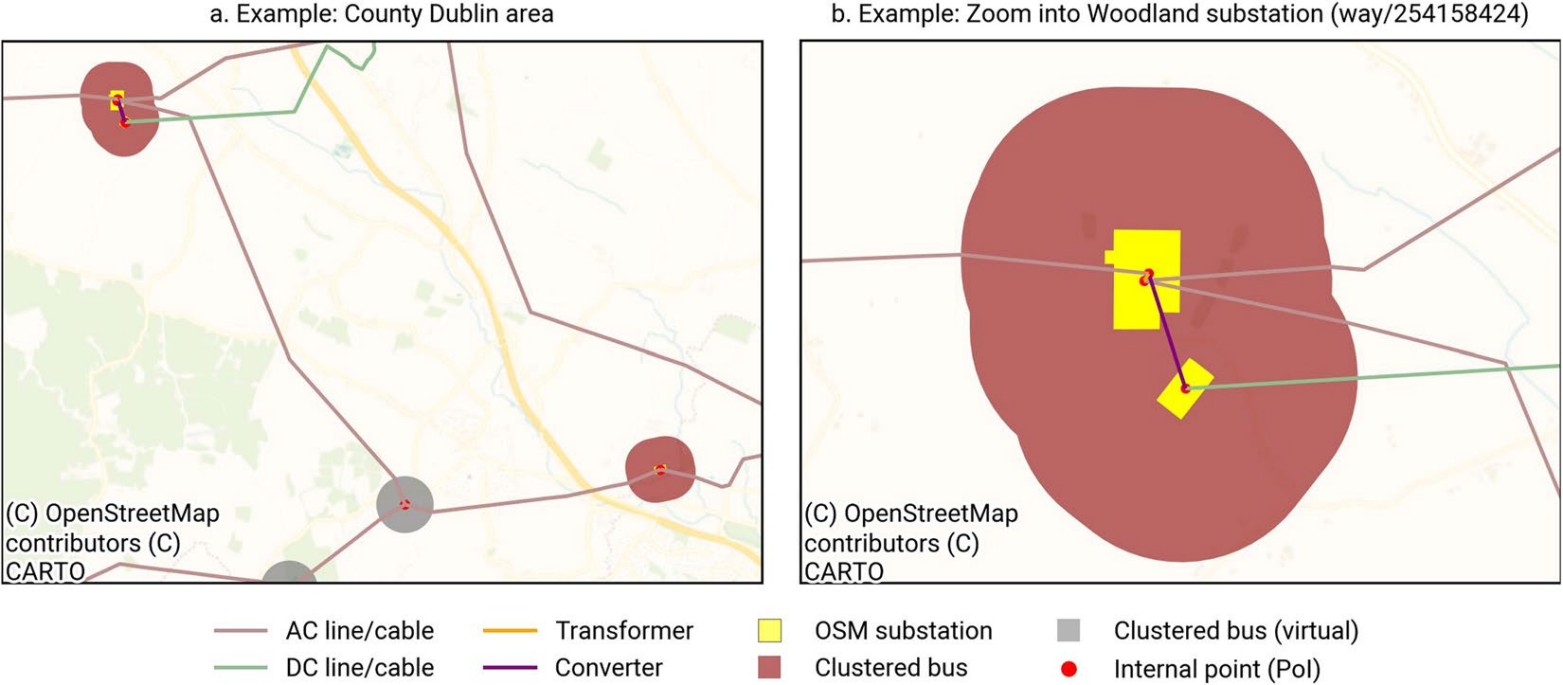
Example of bus clustering. The darkred shape represents the union of the buffer around virtual buses and an original OSM substation polygon (yellow). The bright red dot represents the internal point of the union, this point sets the geographic coordinates of the obtained bus. Lines and cables are connected to the respective voltage level within the substation, transformers are added to connect buses of different voltage levels.

First, we add the endpoints of both AC and DC lines to the set of buses (Fig. 4a). We refer to the buses created from endpoints as *virtual buses*. This step ensures that each line has two corresponding buses to which it is initially connected. Note that this may introduce potential duplicates of buses beyond those already present — which we will take care of later. Next, we specifically consider long lines that are overpassing (multiple) substations. Here we assume that in reality, the substation is electrically connected to the overpassing line. To achieve this, we split the original line into the subsegments between the intersecting nodes and add a suffix to its original identifier (Fig. 4b), to (i) preserve the uniqueness of each line and (ii) still allow for tracing back the line on OSM or OpenInfraMap^9^. We update the endpoints of the split lines, accordingly. For each virtual bus, we evaluate the number of connected lines. If a single line enters and exits the same virtual bus with identical electrical parameters — such as voltage level and number of circuits — the two lines are merged, and the connecting virtual bus is removed. This step simplifies the network by reducing the number of virtual buses, which do not correspond to real substations defined by OSM substation polygons. The remaining virtual buses have an important role in maintaining the network’s topological connectivity, serving as line junctions, such as branching points that directly link multiple transmission lines (see Fig. 5, grey polygon).

Third, we cluster all buses within a radius of 500 m: From each substation polygon provided by OSM and each virtual bus, a buffer of 500 m is created (see Fig. 5). Substations and virtual buses are assigned to the same station identifier and clustered if they overlap. Then we calculate an internal point based on Pole of Inaccessibility (PoI)^36^ which provides the geographic coordinates of the simplified substation. With this methodology, we can always find a polygon-internal point, even if the shape is non-convex. If the clustered shape contains one or multiple OSM substations, the largest substation determines the poi of the simplified bus. Connected lines are remapped, accordingly (Fig. 4d). Finally, we ensure that all given components are correctly electrically connected, i.e., AC buses of different voltage levels at the same substation are linked by transformers, DC buses which represent endpoints of DC lines are connected to the closest AC bus through converters (Fig. 4e) of equal nominal rating, respectively. Figure 5 shows an example how the clustering algorithm is applied and how lines are connected.

The proposed methodology is an enhanced version of the data processing integrated in the PyPSA-Earth model^11^ which has previously demonstrated the potential of using OSM data for global energy system modelling. Our methodology improves the original implementation in efficiency, computational performance and quality regarding the representation of the European high-voltage grid (see Technical Validation Section).

### Step 4 — Creating a PyPSA-ready base network

In a final step, we create a network ready for modelling within PyPSA and PyPSA-Eur. The advantage of our dataset is that it is provided in .csv format, making it easily readable. Further, we include all original and mapped technical parameters of the grid components. Hence, it may potentially be used outside our given use case, directly or with small adaptations.

Based on the voltage levels, we assign each line to a standard line type from PyPSA^12,13,37,38^, selecting the closest matching voltage (e.g. 400 kV is mapped to a 380 kV line type). Using the static grid model provided by 50Hertz^3^, we demonstrate in Fig. 18 that this approach is effective for this particular region in the absence of more accurate data. Using geometry line lengths and number of circuits, we calculate electric parameters, such as impedance, reactance and apparent power ${S}_{nom}^{AC}$ (Eq. 1), as these are not contained in the original OSM input data. We point out that, while the mapped standard line type of a 380 kV and a 400 kV is the same, the calculated apparent power differs given the multiplication with their individual voltage levels. We apply a factor of 0.7 to approximate the N-1 security margin (Eq. 2)^12,39^. Note that this factor can be individually set in the configuration file of PyPSA-Eur. We provide an overview of all resulting AC lines and cables in Table 8.

1 $${S}_{nom}^{AC}={n}_{circuits}\cdot \sqrt{3}\cdot {U}_{nom}^{OSM}\cdot {I}_{nom}^{pandapower}$$

2 $${S}_{n-1}^{AC}=0.7\cdot {S}_{nom}^{AC}$$

**Fig. 6:**
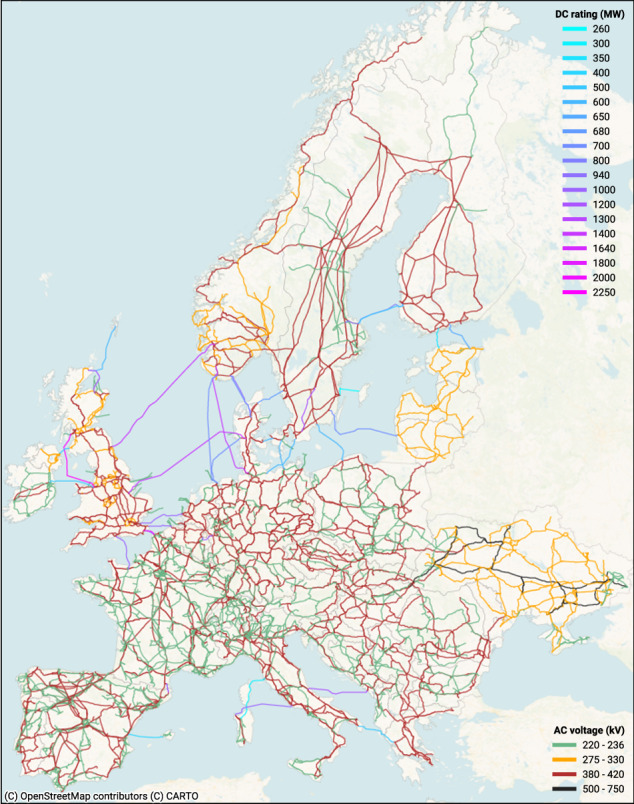
Map of the OSM-based European high-voltage grid. This map was generated using the grid dataset provided with this publication^31^.

**Fig. 7:**
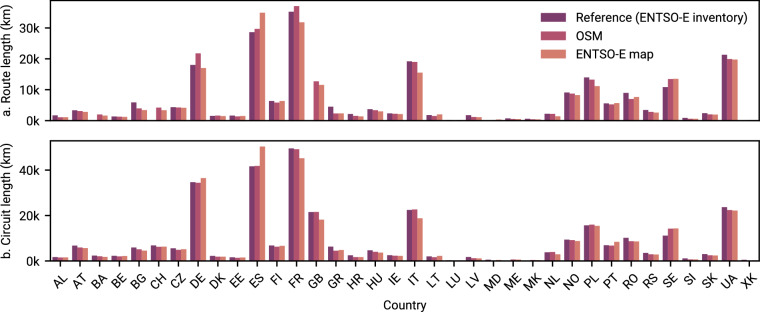
Comparison of total route and circuit lengths per country.

**Fig. 8:**
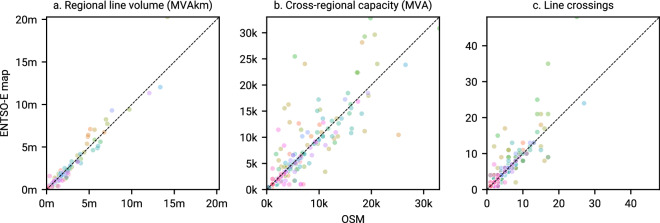
Comparison of line volume per NUTS1 region — Colors represent individual countries. Line volume is the product of the nominal capacity and the length, summed over all lines within the region.

**Fig. 9:**
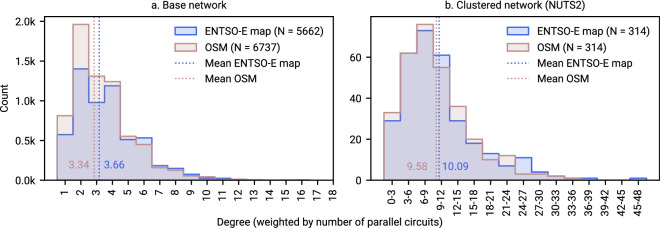
Comparison of the weighted degree distribution in both transmission grid representations before and after clustering (NUTS2). Ukraine at geoBoundaries^48^ administration level 1, Moldova at full bus resolution. A comparison at NUTS3 resolution is provided in Fig. 12.

**Fig. 10:**
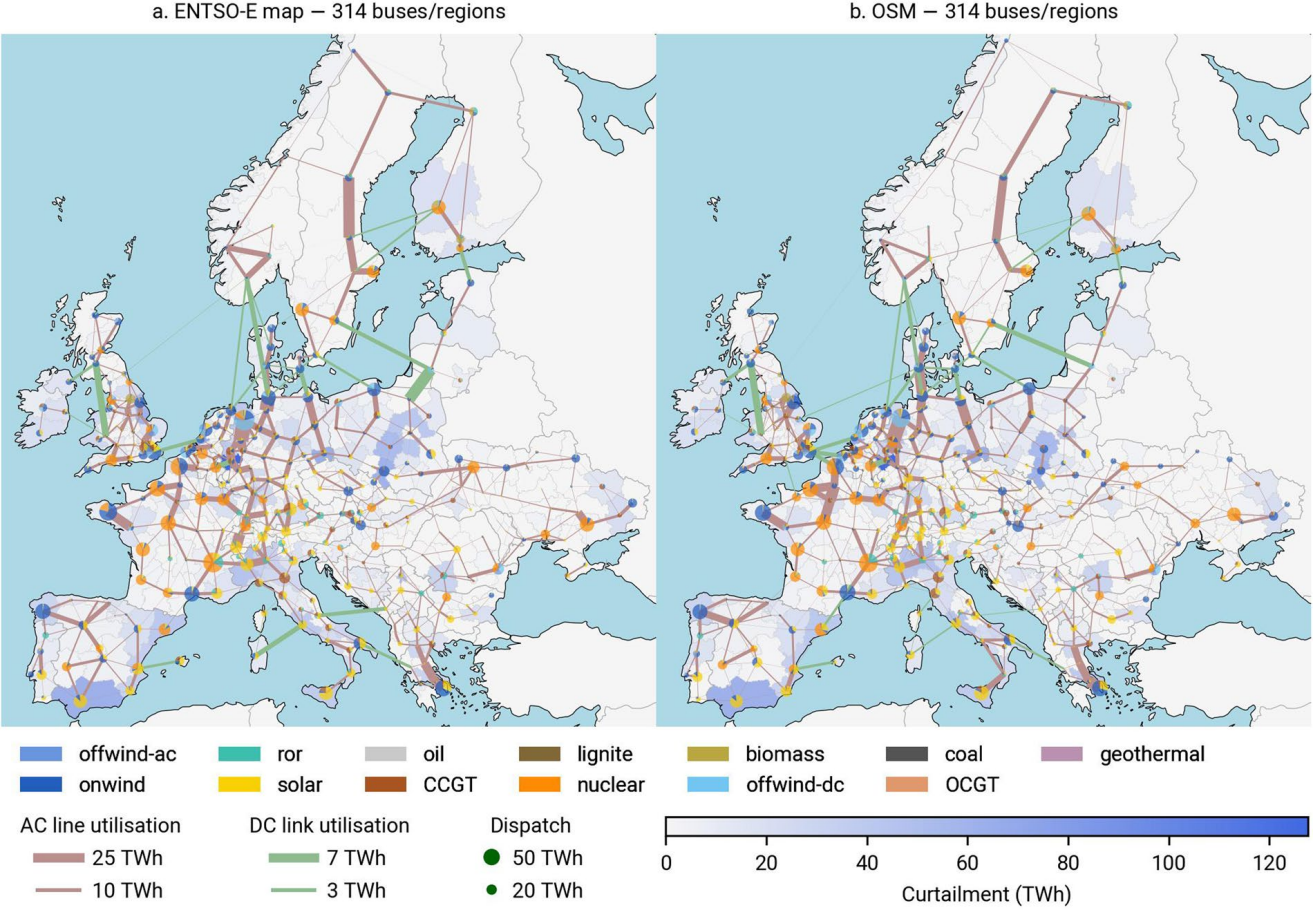
Regional dispatch, line utilisation and curtailment. A map comparing nominal ratings of the two clustered grids is provided in the Fig. 13.

**Fig. 11:**
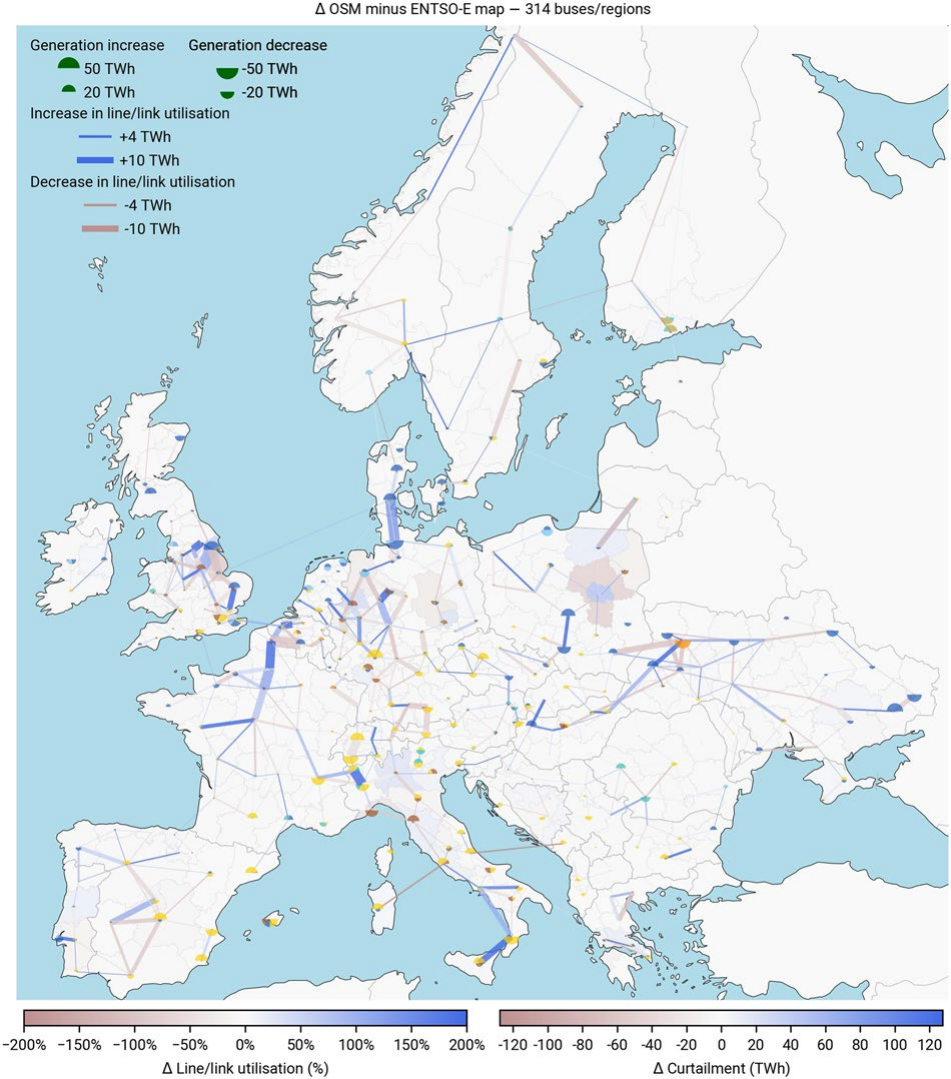
Regional dispatch, line utilisation and curtailment (delta). Blue indicates an increase in curtailment or line utilisation from the ENTSO-E map to the OSM-based transmission grid, while red indicates a decrease. For full transparency, note that this map shows an outer join of all transmission grid elements, including lines and links that are not present in the other network.

For DC links, we use the provided length and nominal rating, directly. We assume that all DC links can be operated in both directions. Lastly, after transformers and converters have been added to the network, we remove all unconnected or islanded components.

## Data Records

The compiled representation of the European high-voltage grid (Fig. 6) is hosted online in .csv format and can be downloaded via the Zenodo repository^31^. The dataset includes the geographical scope of the ENTSO-E member states (without Cyprus, Iceland, and Turkey), i.e., Albania (AL), Austria (AT), Belgium (BE), Bosnia and Herzegovina (BA), Bulgaria (BG), Croatia (HR), Czech Republic (CZ), Denmark (DK), Estonia (EE), Finland (FI), France (FR), Germany (DE), Greece (GR), Hungary (HU), Ireland (IE), Italy (IT), Kosovo (XK), Latvia (LV), Lithuania (LT), Luxembourg (LU), Moldova (MD), Montenegro (ME), Netherlands (NL), North Macedonia (MK), Norway (NO), Poland (PL), Portugal (PT), Romania (RO), Serbia (RS), Slovakia (SK), Slovenia (SI), Spain (ES), Sweden (SE), Switzerland (CH), Ukraine (UA), and the United Kingdom (GB).

We continuously update the dataset as the underlying OSM input data and the workflow improve. As of the submission of this paper, the resulting network (Fig. 6) contains 6737 buses–of which 4931 are original OSM substations polygons and 1806 are virtual buses, 8994 AC lines and cables, 37 simplified/aggregated DC links (and converters at their endpoints, respectively), and 875 simplified AC transformers, comprising a total of 283974 km in total route length. In the following, we describe the structure, parameters, and units of the grid components which are stored as comma-separated value files of the same name. The description refers to the dataset version that forms the basis of this publication (i.e., version 0.6)^31^.

### Buses (buses.csv)

All AC and DC substations are represented as buses and stored in the ‘buses.csv’ file (0.9 MB). The file contains the following columns and properties:

- **bus_id** (string): Unique identifier of the bus. The name of a bus is derived in two ways: If a OSM relation or way was used to create the bus, the prefix starts with ‘way/‘ or ‘relation/‘, accordingly. As such, the original identifier is preserved. If the bus is a result of endpoints of connected lines or cables (‘virtual buses’), without an underlying OSM, the prefix is the two-letter ISO3166-1 alpha-2 country code, numbered from North to South and West to East. The suffix represents the voltage level of the bus.
- **voltage** (integer): Nominal voltage level of the bus in as extracted from the OSM data or connected lines and cables (in the case of virtual buses).
- **dc** (boolean): Boolean flag indicating whether the bus type is DC (True/t) or AC (False/f).
- **symbol** (string): String that describes the nature of the bus. By design, all buses are substations, hence the symbol is ‘Substation’.
- **under_construction** (boolean): Boolean flag indicating whether the bus is under construction (True/t) or not (False/f). This is needed to differentiate commissioned substations from planned transmission projects when activating extensions of the PyPSA-Eur model. By design, all buses included in this dataset are commissioned and hence not under construction.
- **tags** (string): Additional information on the bus, e.g. the prefix of the bus. This string can be used to access the underlying OSM way or relation, if available.
- **x** and **y** (float): Geographical coordinates of the bus in the WGS84 (EPSG:4326) coordinate system, calculated using the PoI approach^36^.
- **geometry** (shapely Point): Geographical point of the bus in the WGS84 (EPSG:4326) coordinate system. This property provides the ‘x’ and ‘y’ formatted as Point object of the open-source Python library ‘shapely’^40^. It can be imported using shapely.wkt.loads() function.

### AC lines and cables (lines.csv)

All AC lines and cables are stored in the ‘lines.csv’ file. Given the high resolution of the geographic linestrings, its file size is larger (19.7) and makes up the majority of the dataset’s content and size. The file contains the following columns and properties:

- **line_id** (string): Unique identifier of the AC line/cable. The name is directly derived from the underlying OSM way or relation, preserving the original identifier. The suffix includes the voltage level in. Merged lines include a ‘merged_‘ prefix, followed by the OSM identifier of the longest contained line/cable as well as the number of additional lines/cables that were merged.
- **bus0** and **bus1** (string): Reference to the buses at the start (‘bus0’) and end (‘bus1’) of the line/cable, see ‘buses.csv’.
- **voltage** (integer): Nominal voltage level of the line/cable in as extracted from the OSM data.
- **i_nom** (float): Nominal current of the line/cable in kA as mapped using using pandapower’s standard line type library^38^.
- **circuits** (integer): Number of parallel circuits of the line/cable, as extracted from the OSM data.
- **s_nom** (float): Nominal apparent power capacity of the line/cable in MV A as calculated using Eq. 1 based on pandapower’s standard line type library^38^. Note that this value represents the maximum technical capacity (100%) taking into account the number of circuits.
- **r** (float): Line resistance in Ω as calculated using the length of the line/cable and pandapower’s standard line type library^38^.
- **x** (float): Line reactance in Ω as calculated using the length of the line/cable and pandapower’s standard line type library^38^.
- **b** (float): Line susceptance in as calculated using pandapower’s standard line type library^38^.
- **length** (float): The length of the line/cable in as calculated using the linestring of the underlying OSM way or relation, transformed from the WGS84 (EPSG:4326) coordinate system to a metric distance projection, i.e., LAEA Europe (EPSG:3035).
- **underground** (boolean): Boolean flag indicating whether the element is underground (True/t) or overhead (False/f), hence an overhead line or a cable. This information is derived from the OSM data.
- **under_construction** (boolean): Boolean flag indicating whether the line/cable is under construction (True/t) or not (False/f). This is needed to differentiate commissioned transmission lines from planned transmission projects when activating extensions of the PyPSA-Eur model. By design, all lines included in this dataset are commissioned and hence not under construction.
- **type** (string): Type of the line as mapped using pandapower’s standard line type library^38^. This property is the foundation for calculating ‘r’, ‘x’, ‘b’, and ‘s_nom’.
- **tags** (string): Additional information on the line/cable, e.g. the contained OSM way or relation. For merged lines/cables, contained elements are separated by semicolons.
- **geometry** (shapely LineString): Geographical line of the line/cable in the WGS84 (EPSG:4326) coordinate system. This property contains geographically highly spatially resolved linestring of the OSM way or relation. The endpoints have been cleaned to exactly end in ‘bus0’ and ‘bus1’. The property is provided as a shapely LineString object^40^. It can be imported using shapely.wkt.loads() function.

### DC lines and cables (links.csv)

All DC lines and cables are stored in the ‘lines.csv’ file (0.4 MB). The file contains the following columns and properties:

- **link_id** (string): Unique identifier of the DC line/cable. The name is directly derived from the underlying OSM way or relation, preserving the original identifier. The suffix includes the voltage level in and a ‘DC’ identifier.
- **bus0** and **bus1** (string): Reference to the buses at the start (‘bus0’) and end (‘bus1’) of the line/cable, see ‘buses.csv’.
- **voltage** (integer): Nominal voltage level of the line/cable in as extracted from the OSM data.
- **p_nom** (float): Nominal real power capacity of the line/cable in as provided by the OSM relation.
- **length** (float): The length of the line/cable in as calculated using the linestring of the underlying OSM way or relation, transformed from the WGS84 (EPSG:4326) coordinate system to a metric distance projection, i.e., LAEA Europe (EPSG:3035).
- **underground** (boolean): Boolean flag indicating whether the element is underground (True/t) or overhead (False/f), hence an overhead line or a cable. This information is derived from the OSM data.
- **under_construction** (boolean): Boolean flag indicating whether the line/cable is under construction (True/t) or not (False/f). This is needed to differentiate commissioned transmission lines from planned transmission projects when activating extensions of the PyPSA-Eur model. By design, all lines included in this dataset are commissioned and hence not under construction.
- **tags** (string): Additional information on the line/cable, e.g. the contained OSM relation.
- **geometry** (shapely LineString): Geographical line of the line/cable in the WGS84 (EPSG:4326) coordinate system. This property contains geographically highly spatially resolved linestring of the OSM way or relation. The endpoints have been cleaned to exactly end in ‘bus0’ and ‘bus1’. The property is provided as a shapely LineString object^40^. It can be imported using shapely.wkt.loads() function.

### Transformers (transformers.csv)

All transformers are stored in the ‘transformers.csv’ file (0.1 MB). The file contains the following columns and properties:

- **transformer_id** (string): Unique identifier of the transformer. The prefix is determined by the substation where the transformer is located. The suffixes contain the voltage ‘bus0‘ and ‘bus1‘ in kV.’
- **bus0** and **bus1** (string): Reference to the buses at the start (‘bus0’) and end (‘bus1’) of the line/cable, see ‘buses.csv’.
- **voltage_bus0** and **voltage_bus0** (integer): Voltage level at the ‘bus0’ and ‘bus1’ side of the transformer in kV, respectively.
- **s_nom** (integer): Rounded (ceiling) apparent power of the transformer in. This is determined by the maximum throughput of the transformer, i.e., the maximum of the sum of connected lines/cables on either side.
- **geometry** (shapely LineString): Geometry of the transformer, represented by a linestring in (EPSG:4326) coordinate system, connecting two buses of different voltage levels at a substation. It can be imported using shapely.wkt.loads() function.

### Converters (converters.csv)

All converters are stored in the ‘converters.csv’ file (0.01 MB). The file contains the following columns and properties:

- **converter_id** (string): Unique identifier of the converter. The suffix is determined by the substation where the converter is located. The suffixes contain the voltage ‘bus0‘ and ‘bus1‘ in kV.’
- **bus0** and **bus1** (string): Reference to the buses at the start (‘bus0’) and end (‘bus1’) of the line/cable, see ‘buses.csv’.
- **voltage**: Maximum voltage level of the converter in kV as extracted from OSM data of the connected DC lines/cables.
- **p_nom** (integer): Nominal real power capacity of the converter in MW, determined by the sum of real power capacities of the connected DC lines/cables.
- **geometry** (shapely LineString): Geometry of the converter, represented by a linestring in (EPSG:4326) coordinate system, connecting an AC and DC bus at a substation. It can be imported using shapely.wkt.loads() function.

### Interactive map (map.html)

The dataset is accompanied by an interactive map built using the open-source packages geopandas^33^ and folium. The map contains all components of the dataset, i.e., buses, lines, transformers, and converters, their geometries and technical parameters. The map can be opened with any modern browser to visually explore the dataset. Different layers can be individually activated or deactivated — technical properties can be inspected by hovering over or clicking on the components. As the map is self-contained and stores all information of the dataset, it is considerably large at 53.1 MB and can be used offline. We provide a screenshot of the map in Fig. 6.

## Technical Validation

We perform two validation steps for assessing the quality of the dataset. First, we compare the dataset with official inventory statistics provided by ENTSO-E. Second, we compare the results of an representative PyPSA-Eur model instance based on the two high-voltage grid datasets: OSM (presented in Fig. 6) and an extract from the online ENTSO-E map using GridKit tool (referred to as ‘ENTSO-E map’)^10^. This network is has been used by numerous PyPSA-Eur users, and is hence a good reference for comparison. In Fig. 17, we further provide a comparison between a geo-referenced, official dataset by 50Hertz^3^ and the OSM-based grid of the region, respectively.

### Comparison with ENTSO-E statistics and map

Based on ENTSO-E’s 2023 inventory of transmission^41^, we first compare the total route (a) and circuit lengths (b) of AC lines and cables on a per country level (Fig. 7). Note that the inventory does not include all statistics for each country, i.e., route lengths are missing for Bosnia and Herzegovina, Switzerland, and Great Britain, while circuit lengths are missing for Montenegro and North Macedonia. While Ukraine and the Republic of Moldova have joined ENTSO-E on 1 January 2024 and 22 November 2023 as full and observing members, respectively, their inventory are not yet included in the dataset. For these two countries and Kosovo, we take reference data from third party sources^42–45^.

We find that our transmission grid based on OSM data is in agreement with the ENTSO-E inventory. Calculating the Pearson correlation coefficient for both route and circuit lengths between the official statistics and the respective transmission grid representations, we see an overall improvement from the ENTSO-E map (${\rho }_{routes}=0.9489$ and ${\rho }_{circuits}=0.9862$) to OSM (${\rho }_{routes}=0.9575$ and ${\rho }_{circuits}=0.9980$) in the reproduction of the high-voltage grid (220 to 750 kV). One of the key reasons for these improvements is the much higher level of geographic detail of lines and cables in the OSM-based transmission grid compared to the stylised lines on ENTSO-E’s interactive map. We observe larger discrepancies for Sweden, where both transmission grid representations seem to overestimate the total lengths of the inventory.

As another validation step, we compare the total line volume on NUTS1 region level (Fig. 8a). Here we see strong similarities between the OSM-based and the extracted transmission grid of the ENTSO-E map, with few outliers (${\rho }_{MVAkm}=0.9674$). While a higher geospatial resolution of lines of the OSM-based transmission grid may contribute to the increase in line volume on average, we calculate ${S}_{nom}^{AC}$ using the more differentiated voltage levels given in the OSM data (see Table 8) as opposed to the clustered voltage levels given in the ENTSO-E map, i.e., 220 kV, 300 to 330 kV, 380 to 400 kV, 500 kV, and 750 kV. This may lead to a more accurate representation of the line volume in the OSM-based transmission grid.

To assess the transmission capacity across regions, we compare the capacity (Fig. 8b) and number of line crossings (Fig. 8c) per NUTS1 border (Fig. 8b). While the two transmission grids strongly correlate, we observe notable differences at individual borders (${\rho }_{MVA}=0.8441$), the same is true for the absolute number of line crossings (${\rho }_{crossings}=0.8575$). Due to different quality in geospatial information contained in both transmission grid representations, buses in one may be offset (or not even exist) in the other. Stronger outliers can primarily be traced back to buses close to NUTS1 borders (Fig. 8b). Notable outliers are located in Spain (light green), in Ukraine (pink), and southwestern parts of Germany (ocher). The reasons for discrepancies can be manifold, e.g. due to differences in exact locations of boundary nodes, missing, outdated or wrong data. In the case of Spain, the main reason for the large discrepancy lies in the representation of the high-voltage grid in and around the Madrid area, where the ENTSO-E map is stylised for clarity. A comparison with an official map provided by the National Geographic Institute of Spain^46^ confirms the OSM topology to be more accurate.

### Comparison of model results

In order to assess the impact of the new transmission grid representation, we compare the results of a representative PyPSA-Eur model run based on the OSM dataset to a run based on the ENTSO-E map which is currently being used in PyPSA-Eur^12^. Note that the results shown in this publication are based on version 0.6 of the released prebuilt high-voltage grid representation on Zenodo (10.5281/zenodo.14144752)^31^. We use the same model setup and input data in both model runs, except for the grid representation. We focus on the electricity sector, taking techno-economic assumptions projected for the year 2030 based on version 0.9.2 of the PyPSA technology dataset^47^. We allow for capacity expansion in renewable energy as well as gas-fired generation capacities. To narrow down the effect of the transmission grid, we do not allow for grid expansion and disable dynamic line rating. We set the carbon price to 100 € per tonne of CO_2_ emitted.

As the number and exact locations of buses differ, we cluster the networks to make them comparable (N = 318 regions/buses). In energy system modelling, clustering is often motivated by the spatial reduction of the optimisation problem. While oftentimes clustering algorithms based on grid topology or resource class are used^48^, we are interested in the regional differences of the two grid representations. As such, we map the buses of both grids to clusters based on administrative boundaries, i.e., NUTS2. For non-NUTS countries such as Ukraine, we use the administration level 1 (geoBoundaries^49^) and for Moldova we keep the full high-voltage substation resolution, i.e., 8 nodes. This yields 318 regions/buses for each of the clustered high-voltage grids, respectively. Note that the clustering process in PyPSA-Eur involves a transformation of all transmission lines and cables to the default voltage level of 380 kV. We then run the model at hourly resolution of the year 2030, yielding 8760 time steps.

Figure 9 compares the weighted degree distribution for the two network topologies. We weight the degree by the number of parallel circuits (Eq. 3) to account for potential different representations of lines and links connecting the same two buses (e.g., single lines with multiple number of circuits or multiple lines with single circuits). If $G=(V,E)$ is a weighted graph with vertex set *V* (buses) and edge set *E* (lines, links, converters, and transformers), and each edge $e\in E$ has a weight $w(e)$, then the weighted degree of a vertex $v\in V$ is given by:

3 $${d}_{w}(v)=\sum _{e\in {\rm{IncidentEdges}}(v)}w(e)$$

where ${\rm{IncidentEdges}}(v)$ is the set of edges incident to *v*. We find that the two base networks have a similar weighted degree distribution (Pearson correlation coefficient ${\rho }_{degree,base}=0.9838$). Notably, the OSM-based transmission grid demonstrates a higher number of buses with degree 2. Clustering the two networks before running the optimisation problem will increase the Pearson correlation coefficient to ${\rho }_{clustered,base}=0.9888$ (using binned data)–indicating that at NUTS2 resolution, the two networks are very similar in terms of connectivity.

**Fig. 14:**
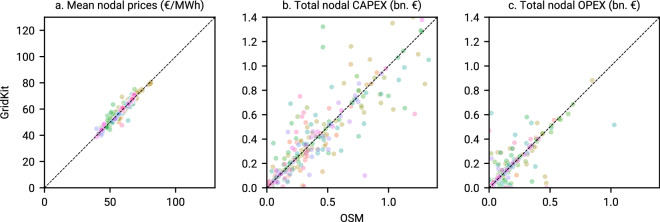
Main results — CO_2_ price: 100 €/t. Comparison of nodal prices, capital expenditures (CAPEX) and operational expenditures (OPEX).

**Fig. 15:**
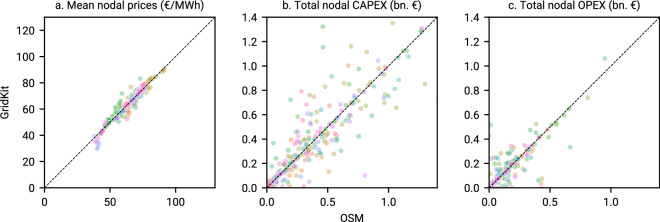
Sensitivity run — CO_2_ price: 200 €/t. Comparison of nodal prices, capital expenditures (CAPEX) and operational expenditures (OPEX).

**Fig. 16:**
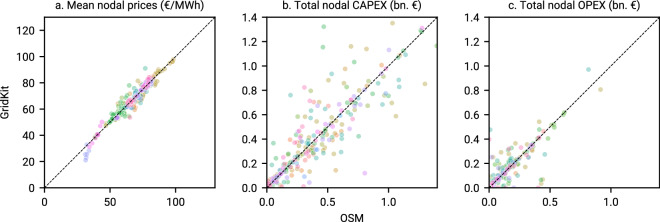
Sensitivity run — CO_2_ price: 300 €/t. Comparison of nodal prices, capital expenditures (CAPEX) and operational expenditures (OPEX).

**Fig. 17:**
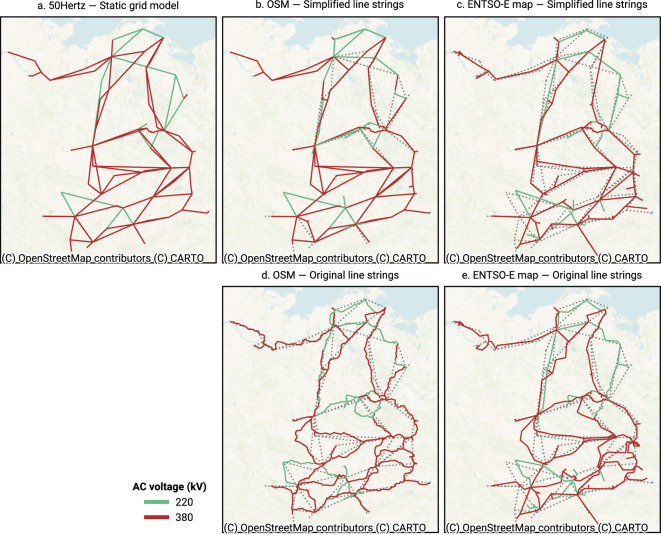
Comparison of OSM and ENTSO-E map-based transmission grid with reference 50Hertz static grid model^3^. Dashed grey lines underneath show the 50Hertz static grid model for comparative purposes. Note that the geospatial data is provided in simplified, point-to-point form, only. Many of the dashed grey lines are also included in the OSM or ENTSO-E map-based grid representations, however, intermediary buses may exist along the line. As such, in their simplification they are not simplified to the level of the 50Hertz static grid model.

**Fig. 18:**
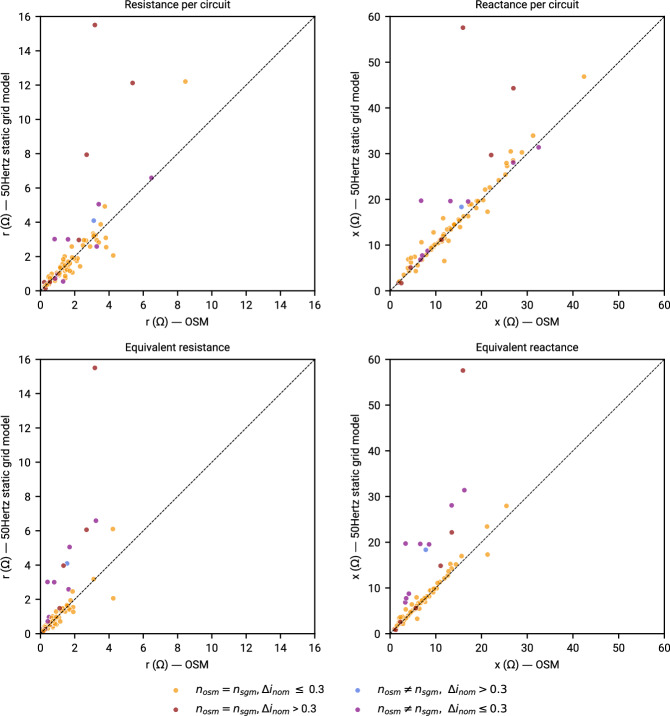
Comparison of AC line/cable resistance and reactance between the OSM-based transmission grid and reference 50Hertz static grid model^3^. ${n}_{osm}$ and ${n}_{sgm}$ refer to the number of parallel circuits for a distinct line in each network, while $\Delta {i}_{nom}=\frac{|{i}_{nom,osm}-{i}_{nom,sgm}|}{{i}_{nom,sgm}}$ refers to the relative change in underlying nominal current.

We solve the two optimisation problems on a high-performance cluster (AMD EPYC 7543 32-Core processor) using up to 130 GB of memory. Each problem takes up to 280 iterations to converge, translating into 20 hours. Running the model for the two transmission grid representations, we find that regional results align closely, including the dispatch of generation assets, utilisation of lines and curtailment (Fig. 10). This is also true for the aggregated picture (Table 4). Total annual system costs drop from 319.28 bn. € in the ENTSO-E map based run to 318.19 bn. € in the OSM-based optimisation, corresponding to mere 0.34 % in difference. The higher weighted degree for both the base and clustered OSM-based high-voltage grid (Fig. 9) indicate a higher topological connectivity, potentially translating into higher degrees of freedom in the optimisation problem compared to the ENTSO-E map based run. This is confirmed when we look into the line and link utilisation and compare the investments. Since aggregate statistics over a continental area can be deceiving (smooth out errors), we show the differences in regional generation, line and link utilisation as well as curtailment in Fig. 11. Here, we can clearly see that the high-voltage grid in OSM is higher utilised than its ENTSO-E map based counterpart. We can also observe that the ENTSO-E map based model compensates by investing more into generation and storage capacities, i.e., +1.3 GW in offshore wind, +1.5 GW in combined cycle gas turbines, +0.6 GW in solar photovoltaics, and +2 GW in battery storage. We also provide an overview of key model results for sensitivity runs with varying CO_2_ prices (200 and 300 €/t) in Tables 9–10. In Figs. 14–16, we compare average electricity prices, CAPEX and OPEX at nodal level.

Bottlenecks in both model runs are located in the same regions, contributing to an annual curtailment in the range of 2742 to 2149 TWh. More prominent differences in line utilisation are visible in Poland, Sweden, northern France, in the western region of Ukraine, southern and central Spain around the Madrid area, as well as southern Italy.

Overall, the results of the two model runs are very similar, indicating that i) both grids seem to adequately represent reality and ii) the OSM-based transmission grid is a suitable replacement for the ENTSO-E map based grid. The higher utilisation of the OSM-based transmission grid is in line with the higher topological connectivity of the network. The differences in the investment decisions are marginal and can be attributed to the differences in grid topology.

We have demonstrated that the dataset aligns well with official statistics and the ENTSO-E map, and that the results of a representative PyPSA-Eur model instance based on both datasets are highly similar. Its core strengths stem from the high level of geographic detail and the continuous updates to the OSM database in combination with a strong PyPSA-Eur user base. The workflow is completely transparent and the data is openly accessible. Although we cannot guarantee the accuracy of the data, as only TSOs have access to the real grid data, we believe this dataset provides the best publicly available representation of the European high-voltage grid.

## Usage Notes

The published dataset is provided under the ODbL 1.0 licence. Geoinformation is encoded in the WGS84 (EPSG:4326) coordinate system. While the dataset and workflow are provided as part of PyPSA-Eur, they are also suitable for a wide range of other applications, including network analyses, power flow calculations, as well as input for other energy energy system models and frameworks. Note that further validation and testing is needed for purposes outside the original scope of this work.

- In order to reproduce the network of this publication, the configuration file ‘config.yaml’: needs to be set to base_network = ‘OSM-prebuilt’, OSM-prebuilt-version: 0.6 and the command snakemake base_network -call needs to be executed.
- To rebuild the network from scratch, this setting can be changed to base_network = ‘OSM-raw’, followed by the command snakemake prepare_OSM_network_release -j 4.
- Per default, buses within the perimeter of a 500 m radius are merged together. This value can be changed in the script, however this may change the topological connectedness of the obtained network.
- Networks can also be built for specific countries, regions or a subset of the countries within PyPSA-Eur by setting list of countries the configuration file.

## Figures and Tables

**Fig. 12:**
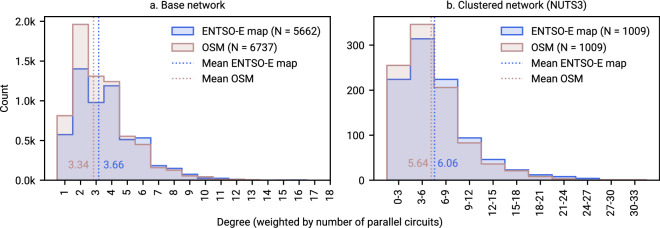
Comparison of the weighted degree distribution in both networks before and after clustering (NUTS3). Ukraine at geoBoundaries^48^ administration level 1, Moldova at full bus resolution.

**Fig. 13:**
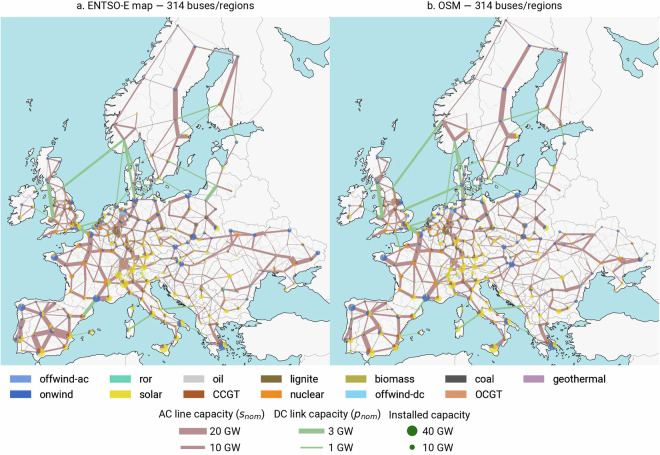
Clustered AC line capacities, DC link nominal ratings, and optimal generation capacities.

**Fig. 19:**
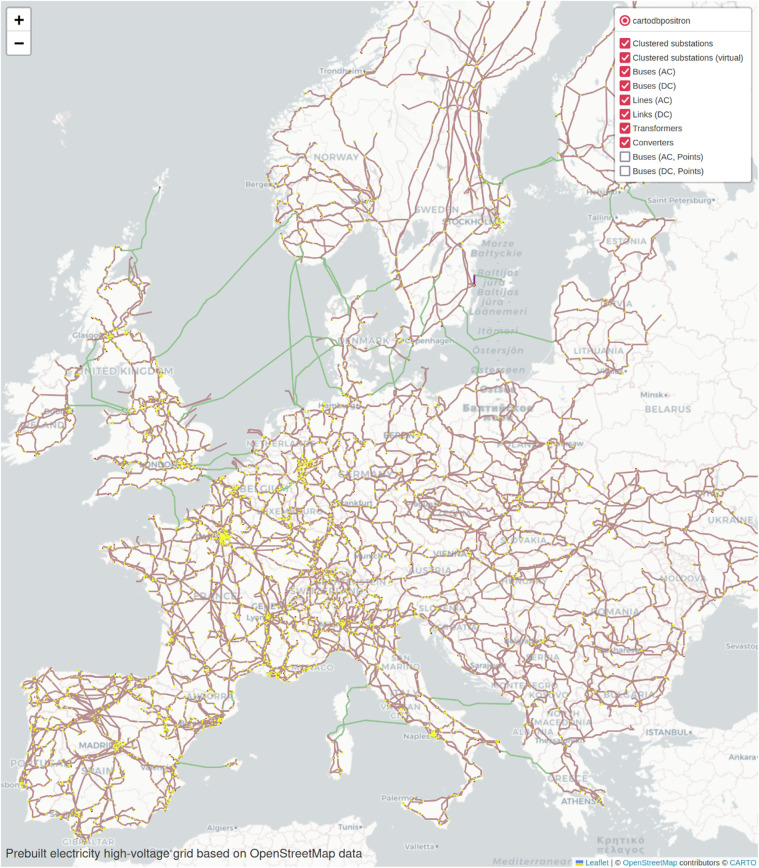
Screenshot of the interactive map visualising the OSM-based transmission grid. The map is included in the dataset released on Zenodo (map.html)^31^.

## Data Availability

The code to replicate the entire workflow and dataset is provided as part of PyPSA-Eur and released as free software under the MIT licence. Different licences and terms of use may apply to the underlying input data.• PyPSA-Eur^12^ on GitHub: https://github.com/pypsa/pypsa-eur• Version 0.6 of the prebuilt network^31^ based on OSM data can be retrieved via the Zenodo repository. This link will also point to future updates: 10.5281/zenodo.14144752• An interactive map is bundled with the dataset on Zenodo^31^ (see Fig. 19). It can also be directly accessed via the PyPSA-Eur documentation^50^: https://pypsa-eur.readthedocs.io/en/latest/data-base-network.html.

## Figures & Tables

**Table 5:** Overpass turbo queries used to extract the high-voltage grid elements from OSM.

Grid element	Overpass turbo query
AC power lines and cables	way[‘power’ = ‘line’], way[‘power’ = ‘cable’] and relation[“route” = “power”][‘frequency’! = ‘0’]
DC links	relation[‘route’ = ‘power’][‘frequency’ = ‘0’]
substations	way[‘power’ = ‘substation’] and relation[‘power’ = ‘substation’]

**Table 6:** Notable projects and datasets modelling the high-voltage grid in Europe (alphabetical order).

Project	Regional scope	Tools/data	Last update	Georeferenced	Data published
50Hertz static grid model^3^	Germany (50Hertz control area)	based on asset inventory	April 2022 (updated once a year)	Yes	Yes
ELMOD^5^	Europe	ENTSO-E interactive map^2^ plus manual changes	January 2014 (data not published)	Yes	No
ELMOD-DE^51^	Germany	VDE, TSO maps, and OSM	March 2016 (single release)	Yes	Yes (not reproducible)
Hutcheon & Bialek^6^	Europe (UCTE plus Balkan region)	PowerWorld model	June 2013 (updated once)	No	Yes (not reproducible)
JAO static grid model^4^	CORE capacity calculation region	based on CORE TSO asset inventory	April 2024 (updated frequently)	No	Yes
PyPSA-Eur^12^	Europe	ENTSO-E interactive map^2^ using GridKit^10^ plus manual changes	January 2022 (updated once)	Yes	Yes (reproduction complex)
osmTGmod^52^	Germany	OSM using Osmosis (SQL and Java)^10^	November 2017 (single release)	Yes	Yes (reproduction complex)
SciGrid (Power)^7^	Europe, Germany	OSM using GridKit^10^	November 2015 (updated once)	Yes	Yes (reproduction complex)
**This publication**	Europe	OSM using Overpass turbo API and Python	November 2024 (updated frequently)	Yes	Yes^31^ (reproducible)

Note that the specific regional scope referring to ‘Europe’ may vary across the listed projects. A comparison of our dataset with the 50Hertz static grid model is shown in Fig. 17.

**Table 7:** List of DC projects in the OSM-based transmission grid.

OSM identifier	DC project name (sorted alphabetically)	From	To	Voltage (kV)	Rating (MW)	Calc. length (km)
relation/8193755	Aachen Lüttich Electricity Grid Overlay (ALEGrO)	BE	DE	320	1000	89
relation/3918230	Baltic Cable	DE	SE	450	600	260
relation/14126301	BritNed	GB-ENG	NL	450	1000	253
relation/8099179	Caithness-Moray Link	GB-SCT	GB-SCT	320	1200	161
relation/17631956	Conexión Mediterránea Transporte Alta tensión (COMETA)	ES	ES	250	400	241
relation/8185420	Copenhagen-Brussels-Amsterdam cable (COBRAcable)	DK	NL	320	700	328
relation/2127794	Cross-Channel (IFA 2000)	FR	GB-ENG	270	2000	69
relation/8184641	East-West Interconnector	IE	GB-WLS	400	500	255
relation/15772117	ElecLink	FR	GB-ENG	320	1000	52
relation/8184630	Estlink 1	EE	FI	150	350	104
relation/6886400	Estlink 2	EE	FI	450	650	174
relation/8184631	Fenno-Skan	FI	SE	400	500	228
relation/3391954	Fenno-Skan 2	FI	SE	500	800	304
relation/3392001	Gotlandslänken (Gotland 2 + 3)	SE	SE	150	260	96
relation/5487095	Great Belt power link	DK	DK	400	600	56
relation/9934065; relation/9934066	Interconnexion Electrique France-Espagne (INELFE)	ES	FR	320	2000	64
relation/10377412	Interconnexion France-Angleterre 2 (IFA-2)	FR	GB-ENG	320	1000	230
relation/9982798	Italy-Corsica-Sardinia (SACOI) — Corsica-Sardinia	FR	IT	200	300	141
relation/3391794	Italy-Corsica-Sardinia (SACOI) — Italy-Corsica	FR	IT	200	300	240
relation/8185664	Italy-Greece	GR	IT	400	500	299
relation/2505320	KONTEK	DE	DK	400	600	166
relation/8184629	Konti-Skan	DK	SE	300	680	142
relation/8185767	Montenegro-Italy (MON.ITA)	IT	ME	500	1200	411
relation/6914309	Moyle Interconnector	GB-NIR	GB-SCT	500	500	61
relation/8185487	Nemo Link	BE	GB-ENG	400	1000	139
relation/9965201	NorNed	NL	NO	450	700	578
relation/8185455	NordBalt	LT	SE	300	700	446
relation/16213216	NordLink	DE	NO	525	1400	620
relation/13295785	North Sea Link	GB-ENG	NO	515	1400	723
relation/8185711	Sardegna-Penisola Italiana (SAPEI)	IT	IT	500	1000	418
relation/15777152	Shetland HVDC Link	GB-SCT	GB-SCT	320	600	282
relation/3391931	Skagerrak (1-3)	DK	NO	350	940	241
relation/8184632	Skagerrak (4)	DK	NO	500	700	235
relation/3392010	SwePol	PL	SE	450	600	254
relation/8184633	SydVästlänken	NO	SE	300	1200	252
relation/15781671	Viking Link	DK	GB-ENG	525	1400	741
relation/15775538	Western HVDC Link	GB-SCT	GB-WLS	600	2250	398

Note that OSM relation identifiers are unique and persistent as long as the object is not deleted. Projects can be directly accessed via the OSM website by clicking on their respective relation identifier in the table.

**Table 8:** Nominal capacities in the OSM base network AC lines and cables to pandapower standard type library.

Voltage (kV)	Line type	Nominal current (kA)	Circuits	Rating (MVA)	Length (km)
220	Al/St 240/40 2-bundle 220.0	1.29	1	492	59805
220	Al/St 240/40 2-bundle 220.0	1.29	2	983	18612
220	Al/St 240/40 2-bundle 220.0	1.29	3	1475	634
220	Al/St 240/40 2-bundle 220.0	1.29	4	1966	79
225	Al/St 240/40 2-bundle 220.0	1.29	1	503	24169
225	Al/St 240/40 2-bundle 220.0	1.29	2	1005	1834
225	Al/St 240/40 2-bundle 220.0	1.29	4	2011	9
236	Al/St 240/40 2-bundle 220.0	1.29	1	527	18
275	Al/St 240/40 3-bundle 300.0	1.935	1	922	3092
275	Al/St 240/40 3-bundle 300.0	1.935	2	1843	1664
300	Al/St 240/40 3-bundle 300.0	1.935	1	1005	4225
300	Al/St 240/40 3-bundle 300.0	1.935	2	2011	30
330	Al/St 240/40 3-bundle 300.0	1.935	1	1106	17107
330	Al/St 240/40 3-bundle 300.0	1.935	2	2212	1016
380	Al/St 240/40 4-bundle 380.0	2.58	1	1698	26384
380	Al/St 240/40 4-bundle 380.0	2.58	2	3396	7802
380	Al/St 240/40 4-bundle 380.0	2.58	3	5094	194
380	Al/St 240/40 4-bundle 380.0	2.58	4	6792	230
400	Al/St 240/40 4-bundle 380.0	2.58	1	1787	79590
400	Al/St 240/40 4-bundle 380.0	2.58	2	3575	18395
400	Al/St 240/40 4-bundle 380.0	2.58	3	5362	13
400	Al/St 240/40 4-bundle 380.0	2.58	4	7150	68
420	Al/St 240/40 4-bundle 380.0	2.58	1	1877	4936
420	Al/St 240/40 4-bundle 380.0	2.58	2	3754	3
420	Al/St 240/40 4-bundle 380.0	2.58	3	5631	12
500	Al/St 240/40 4-bundle 380.0	2.58	1	2234	230
750	Al/St 560/50 4-bundle 750.0	4.16	1	5404	4002

**Table 9:** Sensitivity run — CO_2_ price: 200 €/t.

	System costs (bn. €)	CAPEX (bn. €)	OPEX (bn. €)	Curtailment (TWh)	Generation (TWh)
GridKit	326.83	295.71	31.12	2936.64	3131.02
OSM	325.58	294.66	30.92	2927.38	3128.45
Delta (Percent)	−0.38	−0.36	−0.65	−0.32	−0.08

Comparison of key result metrics between ENTSO-E map and OSM-based transmission grid.

**Table 10:** Sensitivity run — CO_2_ price: 300 €/t.

	System costs (bn. €)	CAPEX (bn. €)	OPEX (bn. €)	Curtailment (TWh)	Generation (TWh)
GridKit	331.81	301.79	30.03	3027.28	3135.37
OSM	330.36	300.83	29.54	3024.33	3132.4
Delta (Percent)	−0.44	−0.32	−1.64	−0.1	−0.09

Comparison of key result metrics between ENTSO-E map and OSM-based transmission grid.

Figure 18 was generated by mapping AC lines and cables of the OSM-based transmission grid to the 50Hertz static grid model (SGM) using OSM tags and SGM names (right join). Note that this data explains 4475 km of 5126 km in route length, as not all lines could mapped. For 79 % of the data, using pandapower’s standard line types^38^ for calculating the resistance and reactance comes close to official data in the SGM (orange). Purple data points show a discrepance primarily due to unequal number of parallel circuits in both datasets (SGM data larger by factor 2). Red and blue data points indicate that underlying line types are entirely different. This is the case for some lines in SGM which are of a newer/stronger 380 kV (allowing higher currents) or weaker 220 kV line type.
